# riboviz: analysis and visualization of ribosome profiling datasets

**DOI:** 10.1186/s12859-017-1873-8

**Published:** 2017-10-25

**Authors:** Oana Carja, Tongji Xing, Edward W. J. Wallace, Joshua B. Plotkin, Premal Shah

**Affiliations:** 1 Department of Biology, University of Pennsylvania, 204K Lynch Labs, 433 S University Ave, Philadelphia, 19104 PA USA; 2 Department of Genetics, Rutgers University, Piscataway, NJ USA; 3 School of Informatics, University of Edinburgh, Edinburgh, EH8 9AB UK; 4 Human Genetics Institute of New Jersey, Piscataway, NJ USA

**Keywords:** Ribosome profiling, Translation quantification, Database, Visualization and comparison tool-kit

## Abstract

**Background:**

Using high-throughput sequencing to monitor translation in vivo, ribosome profiling can provide critical insights into the dynamics and regulation of protein synthesis in a cell. Since its introduction in 2009, this technique has played a key role in driving biological discovery, and yet it requires a rigorous computational toolkit for widespread adoption.

**Description:**

We have developed a database and a browser-based visualization tool, **riboviz**, that enables exploration and analysis of riboseq datasets. In implementation, **riboviz** consists of a comprehensive and flexible computational pipeline that allows the user to analyze private, unpublished datasets, along with a web application for comparison with published yeast datasets. Source code and detailed documentation are freely available from https://github.com/shahpr/RiboViz. The web-application is live at www.riboviz.org.

**Conclusions:**

**riboviz** provides a comprehensive database and analysis and visualization tool to enable comparative analyses of ribosome-profiling datasets. This toolkit will enable both the community of systems biologists who study genome-wide ribosome profiling data and also research groups focused on individual genes to identify patterns of transcriptional and translational regulation across different organisms and conditions.

## Background

Quantification of gene expression using RNA-seq has provided insights into most areas of modern biology [1]. However, ultimately, it is protein synthesis from mRNAs that is responsible for executing most cellular functions. Although mRNA abundance has been used as a proxy for protein production, the correlation between mRNA and protein levels is typically weak and varies widely, likely due to post-transcriptional regulation [2–4]. In contrast, ribosome profiling (**riboseq**) provides a direct method to quantify translation [5, 6]. Ribosome profiling takes advantage of the fact that a ribosome translating an mRNA protects around 30 nucleotides of the mRNA from nuclease activity. High-throughput sequencing of these ribosome protected fragments (called ribosome footprints) offers a precise record of the number and location of the ribosomes at the time at which translation is stopped. Mapping the position of the ribosome-protected fragments indicates the translated regions within the transcriptome. Ribosomes spend different periods of time at different positions, leading to variation in the footprint density along mRNA transcripts. These data provide an estimate of how much protein is being produced from each mRNA [5, 6]. Importantly, ribosome profiling is as precise and detailed as RNA sequencing. Since its introduction in 2009, ribosome profiling has played a key role in driving several biological discoveries [7–26].

Analyses of ribosome profiling datasets can be challenging. In mammalian cells, there can be over 10 million unique footprints. The quantification and processing of these footprints requires computational and domain-specific knowledge.

Despite the similarity between ribosome footprinting and RNA-seq datasets, traditional bioinformatics tools developed for analyzing RNA-seq datasets are limited in their utility when applied to footprinting datasets. For instance, in RNA-seq datasets, variation in distribution of mapped reads along the length of a gene is typically attributed to random sampling. In contrast, several coding sequence features such as biased codon usage, presence of poly-basic amino-acids, and protein-domain architecture affect the distribution of footprinting reads along a transcript [27]. Recently, several tools such as GWIPS-viz [28], RiboGalaxy [29], and RPFdb [30] have been developed for both analysis and visualization of ribosome-profiling datasets. While GWIPS-viz and RPFdb use unified pipelines for processing and mapping footprinting datasets, source code for these tools and the underlying pipelines themselves are not publicly available. As a result, it is difficult to compare the effects of various mapping-related parameters on the overall analyses and visualization. Lack of open source code also limits the use of these tools for analyzing ribosome-profiling datasets in non-model organisms. In addition, tools such as RiboGalaxy and RPFdb are limited by computational resources available on the host servers and can lead to long lag times.

To address these limitations, we have developed an open-source bioinformatics toolkit, **riboviz**, for analyzing and visualizing ribosome profiling data. In implementation, **riboviz** consists of a comprehensive and flexible computational analyses pipeline along with a web application for visualization. The computational pipeline processes raw reads in FASTQ files, trims sequencing adapters, removes rRNA contaminants, aligns reads to ORFs, and generates summary statistics, and metagene and gene-specific QC plots for both RPF and mRNA datasets. Most of the individual steps of the pipeline are parallelized, thereby enabling iterative testing and faster data processing. The visualization tools are based on D3 javascript and R/Shiny and can be set up on any PC.

## Construction and content

### Mapping and parsing riboseq datasets

A major challenge in analyses of ribosome profiling datasets is mapping footprints to ribosomal A, P and E site codons. While several ad hoc rules have been developed to assign reads to particular codons based on the read lengths, these rules are not implemented consistently across studies and as a result, comparing footprinting reads on a gene across datasets remains a challenge. Using a combination of existing tools used for trimming and mapping reads such as *cutadapt* [31], *bowtie* [32], and *hisat2* [33], and custom perl scripts, we have developed a simple set of instructions for mapping reads. We have used this pipeline to remap both RNA-seq and footprinting datasets from published yeast studies to allow comparison of reads mapped to individual genes across different conditions and labs. In addition, researchers can download individual yeast datasets in a flexible hierarchical data format (HDF5) and gene-specific estimates in flat *.tsv* files. The code and documentation for this pipeline are hosted on Github, with a public bug tracker and community contribution (https://github.com/shahpr/RiboViz).

## Utility and discussion

**Fig. 1:**
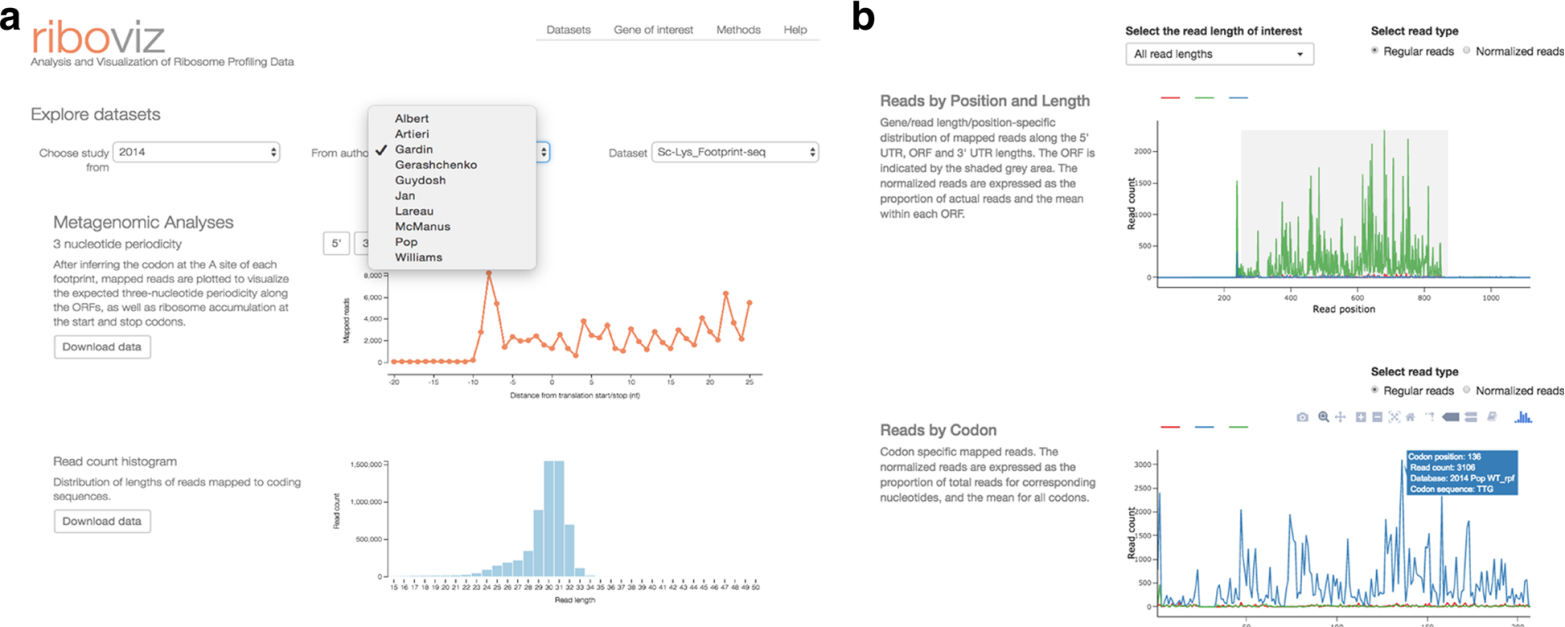
**a** The **riboviz** website with the user interface allowing dataset selection. **b** Distribution of reads mapped to YAL003W in three **riboseq** datasets using a Shiny web server

The web application is available at https://riboviz.org/. Through this web framework, a user can interactively explore publicly available yeast ribosome profiling datasets using JavaScript/D3 [34], JQuery (http://jquery.com) and Bootstrap (http://getbootstrap.com) for metagenomic analyses and R/Shiny for gene-specific analyses. The visualization framework of **riboviz** allows the user to select from available riboseq datasets and visualize different aspects of the data. Researchers can also download a local version of the Shiny application to analyze their private unpublished dataset alongside other published datasets available through the **riboviz** website (Fig. 1).

**riboviz** allows visualization of metagenomic analyses of (i) the expected three-nucleotide periodicity in footprinting data (but not RNA-seq data) along the ORFs as well as accumulation of ribosomal footprints at the start and stop codons, (ii) the distribution of mapped read lengths to identify changes in frequencies of ribosomal conformations with treatments, (iii) position-specific distribution of mapped reads along the ORF lengths, and (iv) the position-specific nucleotide frequencies of mapped reads to identify potential biases during library preparation and sequencing [15, 35–37]. **riboviz** also shows the correlation between normalized reads mapped to genes (in reads per kilobase per million RPKM) and their sequence-based features such as their ORF lengths, mRNA folding energies, number of upstream ATG codons, lengths of 5’ UTRs, GC content of UTRs and lengths of poly-A tails. Researchers can explore the data interactively and download both the whole-genome and summary datasets used to generate each figure.

In addition to the metagenomic analyses, the R/Shiny integration allows researchers to analyze both foot-printing and RNA-seq reads mapped to specific genes of interest, across different datasets and conditions. The Shiny application allows researchers to visualize reads mapped to a given gene across up to nine datasets to compare (i) the distribution of reads of specific lengths along the ORF, (ii) the distribution of lengths of reads mapped to that gene as well as (iii) the overall abundance of that gene relative to its abundance in a curated set of wild-type datasets.

## Conclusions

Ribosome profiling provides a detailed snapshot of translation dynamics within a cell, and has been used to address fundamental questions related to regulation of gene expression in viruses, bacteria, as well as unicellular and multicellular eukaryotes. We have developed a comprehensive analyses and visualization tool – **riboviz** – to enable comparative analyses of ribosome-profiling datasets. This toolkit will enable both the community of systems biologists who study genome-wide ribosome profiling data and also research groups focused on individual genes of interest to identify patterns of transcriptional and translational regulation across different organisms and conditions.
